# Effects of different cardiopulmonary resuscitation education interventions among university students: A randomized controlled trial

**DOI:** 10.1371/journal.pone.0283099

**Published:** 2023-03-14

**Authors:** Yu-Tung Chang, Kun-Chia Wu, Hsiang-Wen Yang, Chung-Yi Lin, Tzu-Fu Huang, Yi-Chi Yu, Yih-Jin Hu

**Affiliations:** 1 Department of Health Promotion and Health Education, National Taiwan Normal University, Taipei, Taiwan; 2 Department of Health Care Management, College of Health Technology, National Taipei University of Nursing and Health Sciences, Taipei, Taiwan; 3 Department of Medical VR, HTC Corporation, Taipei, Taiwan; 4 Taiwan SAVEANNE Education Association, Taipei, Taiwan; 5 Department of Shipping and Transportation Management, National Taiwan Ocean University, Keelong, Taiwan; King Abdulaziz University Faculty of Medicine, SAUDI ARABIA

## Abstract

Cardiopulmonary resuscitation (CPR) education for the public may improve bystander intention to perform CPR on cardiac arrest patients. Studies have shown that different CPR education intervention methods can improve learning performance, with key indicators including attitude toward to CPR, intention to perform CPR, and degree of CPR knowledge and skills. The present study compared the traditional face-to-face method to hybrid and virtual reality (VR) methods to observe difference in learning performance and length of performance retention. This study adopted randomized controlled trial to compare CPR learning performance between traditional face-to-face, hybrid, and VR methods. Participants from each intervention group completed a pretest and 2 posttests. The measurement tools included an attitude and intention questionnaire, knowledge examination, and skill examination with a RESUSCI ANNE QCPR ® manikin. The performance among all participants in pretest showed no significant difference between the intervention groups, indicating no difference in their background attitude, knowledge, and skill level. Significant differences were observed in the average degree of intention to perform CPR between the hybrid and traditional groups in 1st and 2nd posttest. Compared to the pretest results, the posttests revealed significantly higher attitude toward CPR, intention to perform CPR, knowledge examination results, accuracy of overall chest compression, accuracy of CPR procedure, accuracy of AED usage, accuracy of chest compression rate, and accuracy of chest compression depth. The average time to reattending CPR learning and practice session was 11–12 weeks reported by participants. The hybrid and VR methods to CPR education resulted in the same level of improvement in learning performance as traditional face-to-face teaching. The suggested frequency for renewing CPR knowledge and skills is 12 weeks which may be considered in new strategies aimed at promoting CPR education and exposure to the public.

## Introduction

Early activation of “chain of survival” has been proven a key behavior in saving patients from a cardiac arrest [1]. In the steps of the survival chain, activation of emergency response, high-quality cardiopulmonary resuscitation (CPR), and defibrillation are the three most important and fundamental skills that can be performed by bystanders or first responders to a cardiac arrest [1]. Global resuscitation data indicated that survival rates range from 4.6% to 32%, and bystander CPR rates range from 20% to 51% [2–9]. Compared to this global data, Taiwan’s bystander CPR rate is relatively low and should be addressed by first aid educators [5].

CPR education is effective for improving bystander CPR knowledge, attitude, and skill performance [10–18]. In 2015, the American Heart Association suggested that CPR instructors use multimedia, the internet, simulation, and CPR feedback devices to create a hybrid learning environment to improve learning outcomes effectiveness [19]. Studies have indicated that hybrid CPR education approaches effectively improve participant knowledge, attitude, skill, and intention to CPR [13,18]. The retention length of CPR knowledge and skill after CPR education courses has also been considered a measurement of CPR education effectiveness, with previous studies having reported an average retention length of CPR knowledge and skill of 3 months after an education intervention [20–26]. However, there is lack of evidence measuring the results of attitude, and intention to do CPR, and then combining it with length of retention after CPR education.

Approaches to teach CPR include traditional face-to-face, hybrid, and virtual reality (VR) methods [18]. Studies on face-to-face and hybrid courses are well established in the literature, but VR courses are a relatively new style of learning CPR that has only emerged in the past 5 years [18]. Semeraro et al. researched the features of VR in CPR education [27,28]. Since 2019, different types of CPR VR software and applications have been developed by resuscitation councils, universities, and medical education companies [29–37]. However, evidence comparing the overall performance of CPR education approaches with VR is limited.

To compare the effectiveness of different education approaches, the present study investigated two desired outcomes after different methods of CPR education. The first outcome included improvement in knowledge, attitude, skill, and intention to perform CPR after a CPR education course, the second outcome was the length of time until the first outcome was retained.

## Materials and methods

### Study design

This randomized controlled trial (RCT) aimed to exam the CPR performance, attitude toward to CPR, and intention to perform CPR in three teaching methods, including traditional face-to-face, hybrid, and VR methods. Participants underwent a pre-test before CPR education course and two post-tests after CPR education course. There was 1 month interval between 2 post-tests that were held, comprising an attitude questionnaire, knowledge examination, skill examination, and self-reflective feedback about the length of interval between each renewal session. The attitude questionnaire comprised 12 questions measuring the participants’ attitude toward CPR and intention to perform CPR. The knowledge examination included 15 multiple choice questions on topics ranging from basic cardiac physiology, and acute coronary syndrome to CPR and automated external defibrillator (AED) procedures. The CPR skill examination measured the accuracy of CPR and AED steps and CPR performance, including depth, speed, chest recoil, and discontinue time of chest compressions. To be more accurate and consist measure CPR performance, the present study used a RESUSCI ANNE QCPR ® manikin with motor sensor and combined with its software to collect CPR performance data of each session of chest compression.

### Sample size

The sample size was estimated through a statistical power analysis with GPower 3.1.9.7 software, with effect size 0.25, alpha type I error of 0.05, power of 0.8, and 3 groups with 3 times of measurements. The estimated total sample size of all interventional groups was 57. The target population comprised undergraduate and postgraduate students studying in northern Taiwan. The inclusion criteria were over 20 years old, no existing disease, easily stimulated by light and audio, and not holding valid CPR or first aid certification within 2 years. The exclusion criteria were having impaired vision and hearing, undergoing CPR or first aid training, obtaining valid CPR or first aid certification.

### Randomization

**Fig 1:**
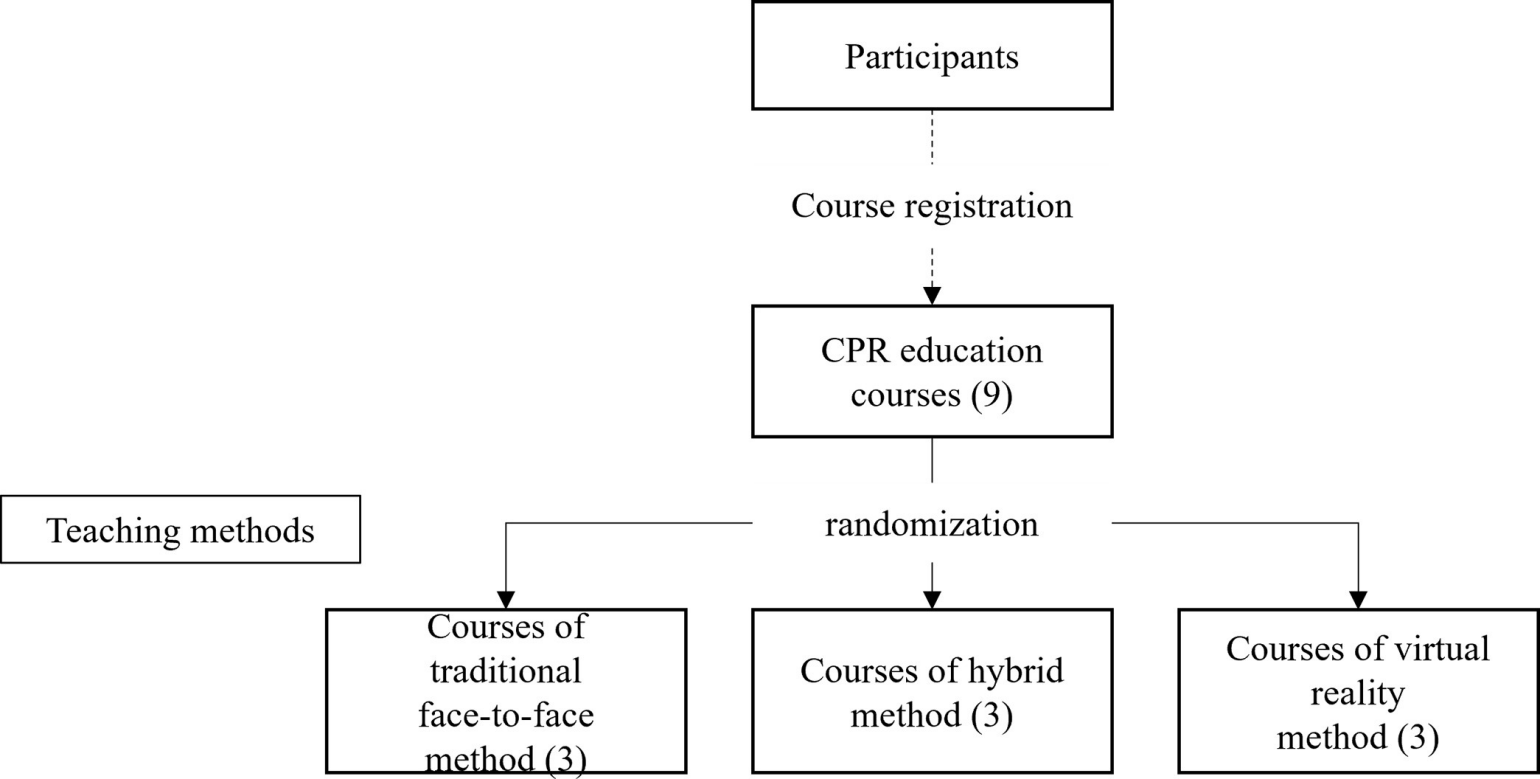
Study randomization.

Those 3 teaching methods were equally adapted in 9 CPR education courses that were available for participants to registration (Fig 1). Participants did not know which teaching methods in each course when they registered. This single-blind approach made participants randomly assigned to each teaching method group.

### Intervention

Teaching methods, including traditional face-to-face, hybrid, and VR methods, were the main interventions in this study. In order to minimize the effects of different teaching methods, the agenda of each intervention were set up in the same manner, which included 40 minutes lecture and 70 minutes practice. The difference between each intervention was the approach to deliver lecture and practice session. The traditional face-to-face method delivered face-to-face approach in both lecture and practice. The hybrid method delivered a web-based video playlist as lecture and face-to-face approach in practice session. The web-based video playlist was provided and authorized by one of Taiwanese online first aid training organization called ANNE School. The VR methods delivered both lecture and practice in HTC Basic Life Support VR platform and using HTC Corporation VR goggles. For each CPR course, the standardized instructor to participant ratio was 1:6 in this study. The CPR courses taught both chest compression only CPR and compression with ventilation. The pre- and post-test examined only chest compression only CPR.

### Ethical approval

The present study was reviewed and approved by the Ethical Committee at En Chu Kong Hospital, Taiwan (ECKIRB1101001). Before each CPR course, instructors introduced the purpose and process of this study to all participants, and guided them through all the contents of informed consent form. Participants signed off informed consent form by themselves and submitted to instructors before study commencement. All signed informed consent forms were kept by person-in-charge (PI) of this trail. The confidential data od participants were kept in encrypted hard-drive by PI as well.

### Statistical analysis

Continuous variables are described using the mean and standard deviation (SD). Categorical variables are presented as frequencies and percentages. The mean comparison of continuous variables between groups was computed using analysis of variance (ANOVA). For comparison of repeated measurements within group, the Wilcoxon Sign Rank Test was adopted due to the sample size. A generalized estimating equation (GEE) was employed to examine the effect on learning performance between the different groups and times of measurement. For all results, p < 0.05 was considered statistically significant. Analyses were performed using SPSS 23.

## Results and discussion

### Results

**Fig 2:**
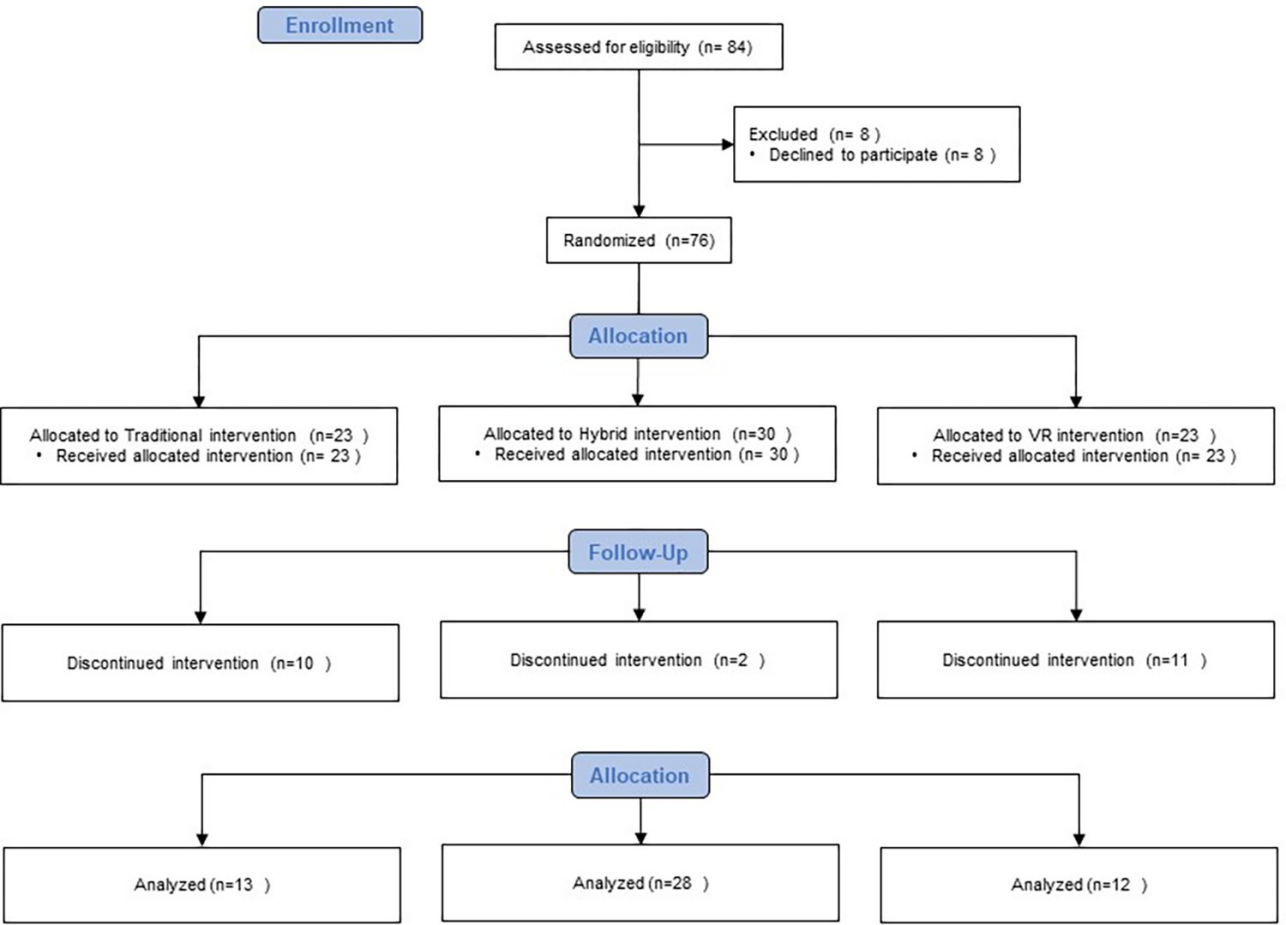
CONSORT diagram for recruitment.

Eighty-four participants were eligibly enrolled into this study. Twenty-three of them were allocated in traditional face-to-face intervention group, 30 were in hybrid intervention group, and 23 were in VR intervention group. (Fig 2)

A total of 76 participants enrolled into this study. The average age was 28.3 years (SD 10.6), with slightly more men (n = 41, 53.9%) than women (n = 35, 46.1%). Among all the participants, 66 (86.8%) reported having no heart disease, 5 (6.6%) had previously received a heart disease diagnosis, and the other 5 (6.6%) were recorded as unknown. For family history, 39 (51.3%) reported having no family history of heart disease, 30 (39.5%) stated that they had a family history of heart disease, and the remaining 7 (9.2%) were recorded as unknown (Table 1).

**Table 1:** Descriptive analysis of participants background between groups.

Groups	Overall (N = 76)		Traditional group n = 23 (30.3%)		Hybrid group n = 30 (39.5%)		VR group n = 23 (30.3%)	
	n (%)	M (SD)	n (%)	M (SD)	n (%)	M (SD)	n (%)	M (SD)
Age		28.3(10.6)		34.4(8.9)		25.0(8.0)		26.4(12.6)
Gender								
Male	35(46.1)		13(56.5)		6(20.0)		16(69.6)	
Female	41(53.9)		10(43.5)		24(80.0)		7(30.4)	
Personal history of heart disease								
No	66(86.8)		22(95.7)		27(90.0)		17(73.9)	
Diagnosed	5(6.6)		1(4.3)		2(6.7)		2(8.7)	
Unknown	5(6.6)		0(0)		1(3.3)		4(17.4)	
Family history of heart disease								
No	39(51.3)		12(52.2)		15(50.0)		12(52.2)	
Diagnosed	30(39.5)		11(47.8)		11(36.7)		8(34.8)	
Unknown	7(9.2)		0(0)		4(13.3)		3(13.0)	

Table 2 summarizes the CPR learning performances results and shows a comparison between and within the groups. The drop-out rate of participants varied from 6% to 45%, which was attributed to a sudden spike in COIVD-19 cases during the study period. The performance among all participants in the pretest showed no significant difference between groups, including that had similar background attitudes (F = 0.38), knowledge (F = 1.49), and skills (F = 1.28). The average degree of intention to perform CPR between the groups differed significantly between the hybrid and traditional groups in both posttests (F = 4.90 and 4.41). Both the traditional and hybrid groups significantly outperformed the better VR group in the 1st posttest results for the knowledge examination, accuracy of overall chest compression, accuracy of CPR procedure, and accuracy of chest compression rate. The traditional group exhibited more accurate chest compression depth than did the VR group in the 1st posttest. Except for intention to perform CPR, all other outcomes differed significantly between the groups at the 2nd posttest. Analysis of the difference between the pretest and posttest outcomes, within groups showed that the performance in the posttests was significantly higher than that in the pretest for attitude toward CPR, intention to perform CPR, knowledge examination results, accuracy of overall chest compression, accuracy of CPR procedure, accuracy of AED usage, accuracy of chest compression rate, and accuracy of chest compression depth. Only the accuracy of chest recoil rate did not differ between the pretest and posttests within all groups.

**Table 2:** Comparison of attitude, intention, knowledge, and performance (between and within groups).

Outcomes	Observations	(A)Traditional group				(B)Hybrid group			(C)VR group		Compare between groups	
		n	M(SD)	Compare within group	n	M(SD)	Compare within group	n	M(SD)	Compare within group	F	Post hoc
Attitude toward CPR	Pretest	23	9.1(0.8)	baseline	30	8.8(0.8)	baseline	22	8.9(1.2)	baseline	0.38	n/a
	1^st^ Posttest	23	9.4(0.6)	-3.08*	30	9.7(0.4)	-4.31*	22	9.5(0.6)	-3.17*	1.23	n/a
	2^nd^ Posttest	13	9.3(0.5)	-1.26	28	9.5(0.4)	-4.12*	12	9.2(0.9)	-2.22*	1.61	n/a
Intention to perform CPR	Pretest	23	6.9(2.1)	baseline	30	7.5(1.5)	baseline	22	7.0(2.4)	baseline	0.71	n/a
	1^st^ Posttest	23	8.5(1.1)	-3.37*	30	9.4(0.8)	-4.70*	22	9.0(0.9)	-3.52*	4.90*	(B)>(A)
	2^nd^ Posttest	13	8.1(1.5)	-2.27*	28	9.1(0.9)	-4.35*	12	8.3(0.8)	-3.06*	4.41*	(B)>(A)
knowledge examination	Pretest	23	61.4(16.3)	baseline	30	67.7(13.1)	baseline	22	62.7(13.2)	baseline	1.49	n/a
	1^st^ Posttest	23	89.2(7.1)	-4.02*	30	88.2(7.9)	-4.54*	22	81.2(12.2)	-3.81*	5.18*	(A)>(C) (B)>(C)
	2^nd^ Posttest	13	79.4(10.3)	-2.75*	28	79.0(10.2)	-3.83*	12	80.5(14.6)	-2.98*	0.07	n/a
Accuracy of overall chest compression	Pretest	23	54.4(32.4)	baseline	30	65.6(32.3)	baseline	22	53.0(29.9)	baseline	1.28	n/a
	1^st^ Posttest	23	97.6(5.6)	-3.95*	30	96.6(5.1)	-4.00*	22	75.9(27.5)	-2.20*	14.5*	(A)>(C) (B)>(C)
	2^nd^ Posttest	13	85.3(20.7)	-2.76*	28	91.2(17.1)	-3.67*	12	87.3(18.9)	-2.51*	0.49	n/a
Accuracy of CPR procedure	Pretest	23	60.0(25.9)	baseline	30	62.3(22.8)	baseline	22	59.5(23.5)	baseline	0.10	n/a
	1^st^ Posttest	23	94.7(10.8)	-3.85*	30	93.6(10.6)	-4.61*	22	83.6(20.1)	-3.02*	4.34*	(A)>(C) (B)>(C)
	2^nd^ Posttest	13	82.3(17.3)	-2.31*	28	85.0(12.9)	-3.73*	12	75.0(22.7)	-2.65*	1.52	n/a
Accuracy of AED usage	Pretest	23	75.5(23.9)	baseline	30	82.0(21.6)	baseline	22	84.0(11.6)	baseline	1.13	n/a
	1^st^ Posttest	23	97.2(7.4)	-3.41*	30	97.0(7.8)	-3.26*	22	93.7(10.7)	-3.23*	1.20	n/a
	2^nd^ Posttest	13	91.3(11.8)	-1.41	28	93.3(7.2)	-1.66	12	97.9(4.8)	-2.33*	2.15	n/a
Accuracy of chest compression rate	Pretest	23	26.2(38.1)	baseline	30	33.2(36.3)	baseline	22	18.4(30.2)	baseline	1.07	n/a
	1^st^ Posttest	23	93.0(11.4)	-3.35*	30	94.5(23.5)	-4.02*	22	49.6(34.0)	-3.50*	22.0*	(A)>(C) (B)>(C)
	2^nd^ Posttest	13	57.7(39.4)	-2.20*	28	74.1(31.2)	-3.55*	12	51.8(36.5)	-1.68	2.12	n/a
Accuracy of chest compression depth	Pretest	23	64.6(41.5)	baseline	30	81.7(35.6)	baseline	22	73.8(38.0)	baseline	1.29	n/a
	1^st^ Posttest	23	99.8(0.4)	-3.51*	30	98.3(6.4)	-2.77*	22	89.5(23.2)	-1.60	4.06*	(A)>(C)
	2^nd^ Posttest	13	90.1(20.0)	-2.70*	28	90.7(22.4)	-2.19*	12	89.0(24.5)	-2.70*	0.02	n/a
Accuracy of chest recoil rate	Pretest	23	78.9(33.5)	baseline	30	72.9(33.4)	baseline	22	75.1(35.4)	baseline	0.19	n/a
	1^st^ Posttest	23	91.8(21.7)	-1.21	30	82.8(30.1)	-1.31	22	83.8(28.6)	-0.50	0.78	n/a
	2^nd^ Posttest	13	86.6(26.9)	-1.32	28	83.8(28.4)	-1.85	12	81.5(29.8)	-1.06	0.10	n/a

*p<0.05.

n/a Post hoc was unnecessary due to no significant difference between groups.

This study collected repeated measurements from each participant. Table 3 summarizes the results from the GEE analysis comparing the effects of CPR learning performance from each group and its interaction with the time variable. The attitude toward CPR, intention to perform CPR, knowledge examination results, and accuracy of CPR procedure showed no significant in the interaction between groups and time that those performance may not change over time. However, the accuracy of overall chest compression and accuracy of AED usage showed that may change in different time points. The figures of comparing each performance between pretest and posttests were provided as supporting information below.

**Table 3:** Analysis of the interaction between CPR education intervention groups and time to CPR learning performance.

	Attitude toward CPR			Intention to perform CPR			Knowledge examination results			Accuracy of overall chest compression			Accuracy of CPR procedure			Accuracy of AED usage		
	Wald χ^2^ Test			Wald χ^2^ Test			Wald χ^2^ Test			Wald χ^2^ Test			Wald χ^2^ Test			Wald χ^2^ Test		
[Group]	0.29			6.60*			1.96			9.16*			3.50			2.63		
[Time]	57.11*			72.08*			193.68*			58.99*			104.68*			47.11*		
[Group]*[Time]	11.85			1.70			8.19			11.08*			2.40			11.13*		
Parameters	B	SE	Wald χ^2^ Test	B	SE	Wald χ^2^ Test	B	SE	Wald χ^2^ Test	B	SE	Wald χ^2^ Test	B	SE	Wald χ^2^ Test	B	SE	Wald χ^2^ Test
[Groups = VR]	-0.16	0.30	0.28	0.48	0.74	0.43	1.28	4.32	0.08	-1.38	9.09	0.02	-0.45	7.22	0.04	1.28	4.32	0.08
[Group = Hybrid]	-0.22	0.22	1.00	0.78	0.59	1.76	6.32	4.07	2.40	11.23	8.80	1.62	2.33	6.69	0.12	6.32	4.07	2.40
[Group = Traditional]	0^a^	.	.	0^a^	.	.	0^a^	.	.	0^a^	.	.	0^a^	.	.	0^a^	.	.
[Time = 2^nd^ Posttest]	0.51	0.23	4.89	1.48	0.50	8.77*	17.67	3.55	24.71*	30.96	7.96	15.11	21.41	5.73	13.94*	17.67	3.55	24.71*
[Time = 1^st^ Posttest]	0.45	0.17	6.94*	1.91	0.42	20.33*	27.82	3.36	68.20*	43.26	6.98	38.30	34.78	5.59	38.61*	27.82	3.36	68.20*
[Time = Pretest]	0^a^			0^a^	.	.	0^a^	.	.	0^a^	.	.	0^a^	.	.	0^a^	.	.
[Groups = VR] * [Time = 2^nd^ Posttest]	0.24	0.13	3.40	-0.04	0.72	0.03	-1.26	5.21	0.05	3.51	11.98	0.08	-6.69	9.12	0.53	-1.26	5.21	0.05
[Groups = VR] * [Time = 1^st^ Posttest]	0.34	0.09	12.95*	0.22	0.65	0.11	-9.35	4.34	4.63	-20.39	11.30	3.25	-10.69	8.19	1.70	-9.35	4.34	4.63
[Groups = VR] * [Time = Pretest]	0^a^	.	.	0^a^	.	.	0^a^	.	.	0^a^	.	.	0^a^	.	.	0^a^	.	.
[Group = Hybrid] * [Time = 2^nd^ Posttest]	0.01	0.34	0.01	0.31	0.59	0.27	-6.53	4.09	2.54	-5.42	10.02	0.29	1.19	7.04	0.02	-6.53	4.09	2.54
[Group = Hybrid] * [Time = 1^st^ Posttest]	0.25	0.21	1.36	0.16	0.52	0.10	-7.38	3.93	3.51	-12.26	9.32	1.73	-3.44	6.61	0.27	-7.38	3.93	3.51
[Group = Hybrid] * [Time = Pretest]	0^a^	.	.	0^a^	.	.	0^a^	.	.	0^a^	.	.	0^a^	.	.	0^a^	.	.
[Group = Traditional] *[Time = 2^nd^ Posttest]	0^a^	.	.	0^a^	.	.	0^a^	.	.	0^a^	.	.	0^a^	.	.	0^a^	.	.
[Group = Traditional] * [Time = 1^st^ Posttest]	0^a^	.	.	0^a^	.	.	0^a^	.	.	0^a^	.	.	0^a^	.	.	0^a^	.	.
[Group = Traditional] * [Time = Pretest]	0^a^	.	.	0^a^	.	.	0^a^	.	.	0^a^	.	.	0^a^	.	.	0^a^	.	.

*p<0.05.

^a^. Set to zero to because this parameter is redundant.

After the intervention, the participants were asked to provide feedback on how frequently they would be willing to attend CPR learning and practice sessions after attending an initial CPR course so they could maintain their knowledge and skill. Overall, the average weeks for re-attending the CPR learning and practice session was 11–12 weeks. This average did not differ significantly between the groups.

### Discussion

Studies have suggested that CPR education intervention may positively affect a bystander’s attitude toward CPR, intention to perform CPR, knowledge, and skill performance [10–18]. Comparing to traditional face-to-face teaching, newly developed methods, such as hybrid and VR methods, show partial improvements in attitude, knowledge, and skill performance [13,18]. The results of the present study also show that CPR education improves CPR attitude, intention to perform CPR, knowledge, and skill which supports the literature. However, the current study is the first to investigate the effectiveness of both the hybrid method and HTC VR method in Taiwan. Although the level of improvement in performance by the experimental groups did not differ significantly from that in the controls, the results still showed a significant improvement after the intervention. New education methods might not necessarily replace existing methods, but they may be ideal options for CPR instructors to implement in circumstances such as the COVID-19 pandemic or where close-contact restrictions apply.

Previous studies have reported that the average retention length of CPR knowledge and skill was 3 months after an education intervention [20–26]. Due to a spike of COVID-19 cases in Taiwan, the present study could only observe the length of retention till 1 month after the intervention. Nonetheless, the results show significant improvement both immediately after the intervention and 1 month after the intervention. It would be ideal to continue any observations after an intervention to more accurately measure the real length of the CPR education effect. Despite this shortcoming in the present study, the participants still provided valuable information about self-evaluation and that the ideal frequency for re-attending CPR learning and practice sessions after CPR course was approximately 12 weeks. Current regulations in Taiwan state that the renewal period for maintaining CPR certification is 2 years [5]. Attending and conducting regular CPR courses every 12 weeks would be time consuming and a cost burden for both the public and CPR instructors. As an alternative approach to increase the frequency of public exposure to CPR knowledge, CPR education institutes and instructors may consider using multimedia channels, social networks, and technologies such as VR to provide key information on CPR. Regarding the frequency of CPR practice sessions for the public, more accessible approaches that meet the 12-week frequency exposure might include short and deliberate practice sessions or CPR practice kiosks in public areas. In the adaptation of new and emerging technologies, VR may also be an option for practicing CPR without having to physically travel to an education site.

Future studies may wish to investigate the length of CPR performance between non-intervention and frequent intervention groups. Examining the effects of newly developed CPR education approaches and VR techniques may reveal ways to improve public accessibility to CPR education during period of global uncertainty, such as with the COVID-19 pandemic. Also, considering the benefit of operating new teaching method and technology may reduce the numbers of educators in each teaching session or can become remotely accessing each session and allow more students to participate.

Two limitations were noted while conducting in this study. First, public fear due to a spike in COVID-19 cases caused a high drop-out rate in the follow-up observation that led to underpower sample size. Second, the target population was composed entirely of university students who may have already been exposed to CPR or first-aid information before. Although the recruitment process followed randomized approach, the discrepancy in gender and age in each group should be improved in further study. Future studies should consider recruiting participants from a more diverse range of backgrounds or age groups.

## Conclusions

In conclusion, different CPR teaching methods were equally effectiveness to improving attitude toward CPR, intention to perform CPR, knowledge, and skill performance after a CPR training intervention. The participants indicated that approximately 12 weeks was the ideal frequency for CPR education exposure. CPR instructors may wish to consider how this affects the delivery of and accessibility to CPR courses for the public.

## Supporting information

S1 Fig Figures for GEE results. (PDF)
