# Supplementary figures and images for: Effects of different cardiopulmonary resuscitation education interventions among university students: A randomized controlled trial
Source: PLoS One. 2023 Mar 14;18(3):e0283099. doi: 10.1371/journal.pone.0283099 (PMC10013893; doi:10.1371/journal.pone.0283099)

Supporting information (figures for GEE results)

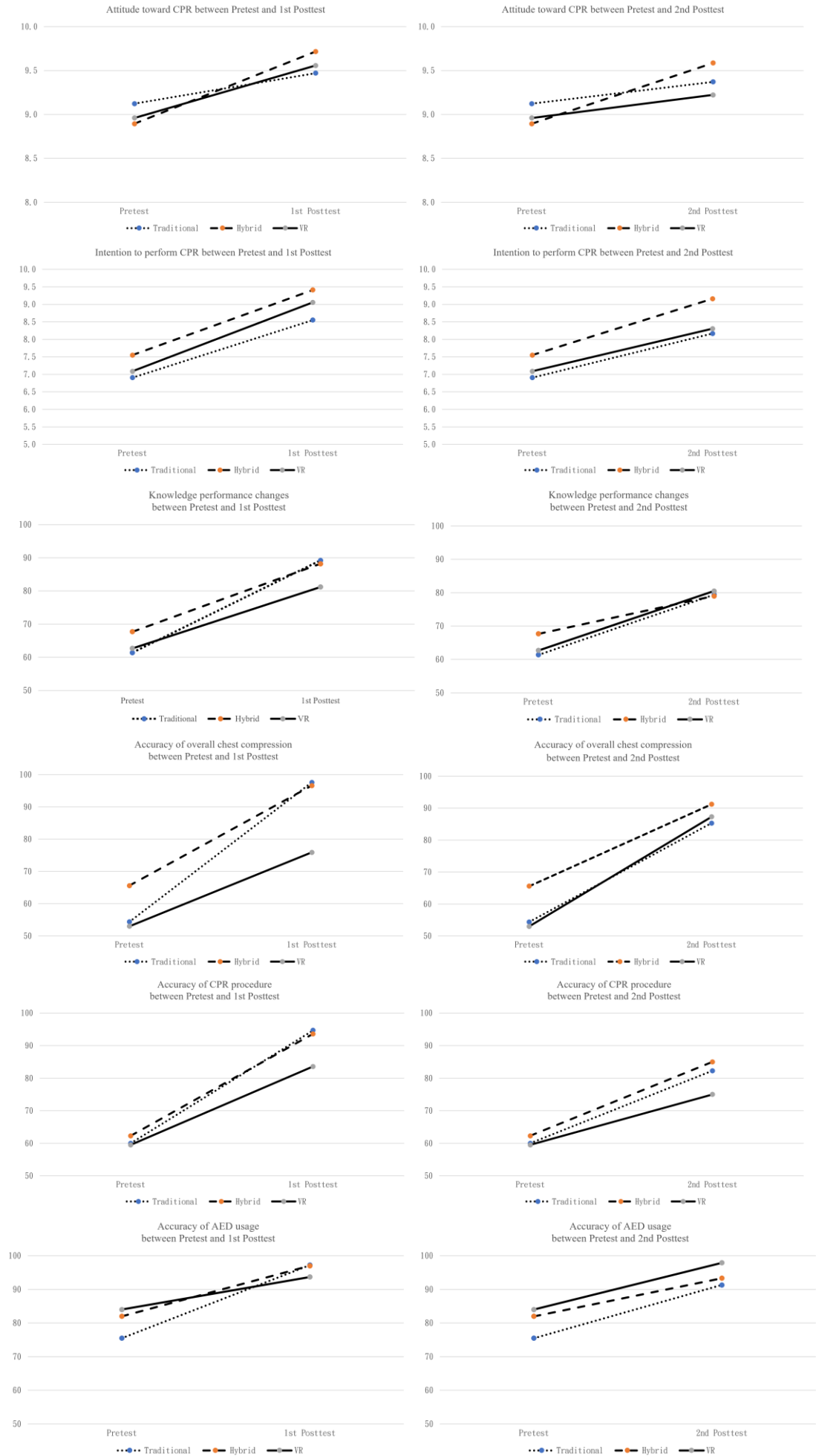

Supplement: S1 Fig — (PDF) [file pone.0283099.s001.pdf]
